# The psychosocial impact of eye-gaze assistive technology on everyday life of children and adults

**DOI:** 10.1080/07853890.2024.2318397

**Published:** 2024-03-05

**Authors:** Maria Andreassen, Maria Borgestig, Helena Hemmingsson

**Affiliations:** a Department of Health, Medicine and Caring Sciences, Linköping University, Norrköping, Sweden; b Department of Women’s and Children’s Health, Uppsala University, Uppsala, Sweden; c Department of Special Education, Stockholm University, Stockholm, Sweden

**Keywords:** Adult, child, complex communication needs, eye-gaze controlled devices, psychosocial impact of assistive devices scale (PIADS)

## Abstract

**Purpose:**

This study investigates the psychosocial impact of eye-gaze assistive technology (EGAT) in both children and adults with long-term experience using eye-gaze assistive technology in everyday life, as well as the psychosocial impact as related to duration of use.

**Methods:**

In this descriptive comparative study, 34 adult and 27 child EGAT users participated in a structured individual interview using the Psychosocial Impact of Assistive Devices Scale (PIADS).

**Results:**

The participants’ age ranged from 5–74 years, 50% were female and 52% had been diagnosed with cerebral palsy. The EGAT had a positive psychosocial impact on competence, adaptability, and self-esteem among adult and child users. Competence was the only subscale with a higher value for adults (*p* = 0.038), compared to children. The items with the highest impact for the psychosocial aspects were quality of life, ability to participate, and self-esteem. The adults had longer duration of use than children, but for high-, medium-, and low-duration users, the device showed a positive psychosocial impact.

**Conclusions:**

Participants considered EGAT to have high positive impacts for participation and quality of life. The study adds new knowledge in that high positive psychosocial impact may be found even among low-duration users of EGAT, which is important to consider for service providers.

## Introduction

Having access to the digital world is, for many people, a part of daily living including work and social interaction [1]. Eye-gaze–controlled computers are high-tech assistive technologies (AT) for individuals who have severe physical disabilities and complex communication needs that do not have the ability to steer a computer manually by switches, or by speech [2]. For those individuals, steering a computer using one’s gaze can be the only possible access method. Through the computer, eye-gaze assistive technology (EGAT) provides individuals access to the digital world at both personal and societal levels.

Research indicates EGAT has the potential to increase accessibility to and participation in computer activities related to play and learning [3–6]. The technology has been found to facilitate communication [3,7,8], increase interactive communication with a partner [9] and increase quality of life (QoL) for individuals with severe impairments such as for example amyotrophic lateral sclerosis (ALS) [7,10]. Findings show the usability of EGAT for individuals who have severe physical disabilities and complex communication needs, and both adults and children have been shown to be quite satisfied with the assistive technology [3,4,7,11]. Nevertheless, at the same time, research shows that the daily use of the EGAT is rather low. A total population study among individuals that had received eye-gaze technology as assistive technology in Sweden demonstrated that 63% used the technology daily and 33% used it weekly with no significant difference between adult and child users. The duration of use per day, however, was higher in adults (age *M* = 41 years) where 45% used the EGAT more than 2 h/day compared to children (age *M* = 12.5 years) where 18% used the EGAT more than 2 h/day [6]. The higher use in adults is supported by [7,8] who report a median use of EGAT of 300 min/day or more in adult users with amyotrophic lateral sclerosis (ALS). The total population study also demonstrated differences in diagnosis between adult and child EGAT users. Although cerebral palsy (CP) was the most common diagnosis in both adults (29%) and children (77%), adult users more often had a diagnosis received after childhood such as (ALS) 26%, or stroke and brain injury 11%, while nearly all children had had their diagnosis since birth. This in turn indicates differences between adults and children in basic reading and writing skills and computer competence when they received the EGAT [6]. In addition, adults and children have quite different lives and priorities, which probably also influence their perception of an AT and their duration of use. For that reason, it might be valuable to examine both groups and compare their perception of EGAT in order to obtain more precise information on their experiences using the technology.

Whether or not a person uses an AT has several reasons. Although AT’s usability is rated as high, personal and contextual circumstances might hinder effective use, and render the technology not worth using from a specific individual’s perspective. Research points to several reasons for abandoning assistive technology (AT), functional reasons as well as psychosocial reasons including the aesthetics of the device [12]. Abandonment of an AT might be related to the personal experience of being an AT user. An individual can, for example, avoid using AT because they consider the use of AT affects their personality by signalling dependence, weakness, and helplessness, which might be something that does not fit with the person’s perception of self [12–14], even if the avoidance results in decreased independence or fewer communication opportunities.

The feelings connected to an AT are deeply personal, and the same AT might give rise to opposite feelings in different users (1). For example, a high-tech assistive device might, for some, be experienced as a sign of modernity and a useful tool, allowing them to participate more fully in valuable activities, and for others it may be seen as a sign of weakness, illness, and helplessness. This, in turn, will influence the individuals’ eagerness to try out and adopt an AT [12,14,15]. The AT in and of itself might also arouse positive and/or negative feelings during its use. Ease of use, functional benefits of using the AT, and how well the AT integrates into everyday life for the person and their surroundings are of importance; as are emotions and psychosocial consequences related to otherness and stigmatization that can influence willingness to use AT [14]. For these reasons, the individual might have mixed feelings when using an AT. For example, steering a computer with the eyes is something that most people do not do, and might be experienced as stigmatizing and attracting unwanted attention. At the same time, gained access to an increased activity repertoire and increased opportunities for participation might have a positive impact on the user’s feeling of competence, happiness, and their quality of life [7,10,16]. For example, found that an AT that positively increases social interaction in children/young people influences their willingness to use it. In summary, an individual might consider how necessary an AT might be to achieve a goal and in improving quality of life in relation to the AT’s shortcomings, such as decreased effectiveness, practicalities, and the risk of negatively influencing their own or others’ perception of them as a person. Therefore, only considering an AT’s functionality and having an outsider’s view of increased opportunities for participation in activities is not enough to understand and predict whether or not an AT will be used [17,18]. The psychosocial impact including the AT’s impact on QoL might be just as important. Investigating the psychosocial impact of an AT on its user may provide valuable information on why an AT is used or not used [17]. It might provide knowledge about which areas are problematic when the AT is used, as well as information on more positive areas. Getting access to the user’s perceptions of an AT is important for the development of the AT in and of itself, as well as the service in relation to the AT [19,20]. In addition, increased knowledge among service providers on psychosocial impact of an AT and its effect on QoL can support clinical reasoning and decision-making in the provision of services to and development of information for EGAT users [19,21]. Investigated stakeholder’s consensus for decision making in eye-gaze technology for children, adolescents and adults with cerebral palsy in order to develop clinical guidance. The results bring up physical, technical, environmental aspects as well as the importance of staff expertise and working in teams. The user’s preferences, interest and goals of using the device is mentioned as important information in relation to the decision to carry out a trial with eye-gaze technology. However, psychosocial aspects per see are rarely brought up by stakeholders which in turn indicate that these aspects might be overlooked.

Over a period of about ten years, a Swedish research group has conducted a series of studies investigating the effect of eye-gaze assistive technology used to steer a computer on participation in computer activities related to play, learning, and communication [3,9,11]. Different stakeholders’ experiences have also been investigated [11,22]. Nevertheless, because these individuals with severe physical disabilities and complex communication needs are not easily interviewed, their personal feelings and the psychosocial impact of using EGAT merit further investigation.

This study investigates the psychosocial impact of eye-gaze assistive technology in both children and adults that have long-term experience using eye-gaze assistive technology in everyday life. In addition, differences in psychosocial impact on children and adult users are investigated, as is the psychosocial impact in relation to the duration of use.

## Method

### Research design

The study is a descriptive comparative study [23], in which 34 adult EGAT users and 27 parents to child EGAT users participated in a structured individual interview using the PIADS. The project received ethical approval from the Regional Ethical Review Board in Linköping, Sweden (study code 2016/218-31).

### Context of the study

Assistive technology, including eye-gaze controlled devices, necessary for daily living is part of Swedish public healthcare and is free-of-charge [24]. If needed, special expertise is available in assistive technology centres located in all 21county councils in Sweden. EGAT devices, used by the participants are specially adapted to the user’s needs. For example, adapted software with communication pages and speech output to communicate with others by eye-gaze. The prescription and use of assistive technology is included as part of the individual’s health record.

### Participants

The current studýs participants were included in a total Swedish population survey study investigating the perceived usability of eye-gaze controlled computers in everyday life. The study addressed the population in Sweden using EGAT, provided by the county council’s assistive technology centre, with a total of 418 users [6]. Of the total population in the survey study, 171 participants (adults, *n* = 111, parents of child users, *n* = 60) agreed to participate and answered the survey. In the survey, a final question asked whether participants agreed to participating in an additional telephone interview about the psychosocial impact of using EGAT in everyday life. Sixty-one participants (adults, *n* = 34, and parents of child users, *n* = 27) agreed to participate in the current study and gave their contact information by telephone or e-mail.

### Demographic data

For the present study, demographic data were retrieved from the total population survey study [6] for those that had agreed to participate in the additional telephone interview. The data retrieved were: participants’ age, gender, diagnoses, communication modalities with other people in the same room, time with access to EGAT (months), whether the EGAT was used at work/school and during leisure, total score from the assessment with the Quebec User Evaluation of Satisfaction with Assistive Technology (QUEST) [25], and duration of use.

### Measurement

The Psychosocial Impact of Assistive Devices scale (PIADS) assesses the psychosocial impact of assistive technology [26]. PIADS relates to 26 various items and the items are divided into three subscales; competence, adaptability, and self-esteem (see Table 1).

**Table 1.** The three subscales in PIADS, description of what the subscale measures, and items included in each subscale.

Subscale:	Competence	Adaptability	Self-esteem
Measures perception of:	Competence and efficacy	Willingness to try out new things	Feelings of emotional health and happiness
Number of items:	12	6	8
List of items:	Adequacy	Ability to participate	Embarrassment
	Capability	Adapt daily living	Frustration
	Competence	Eagerness to try new things	Happiness
	Confusion	Take advantage of opportunities	Security
	Efficiency	Well-being	Self-confidence
	Expertise	Willingness to take chances	Self-esteem
	Independence		Sense of control
	Performance		Sense of power
	Productivity		
	Quality of life		
	Skilfulness		
	Usefulness		

In the interview incorporating PIADS, the participants were asked “Has the EGAT made you/your child feel more or less …(item)?” When needed, the meaning of each item was clarified from a PIADS glossary; for example, ‘embarrassment’ is clarified as “Feeling awkward or ashamed”.

The participants rate the psychosocial impact of the assistive technology for each of the 26 items on a seven-point ordinal scale. The range of scores is from −3 (maximum negative impact) through zero (no perceived impact) to 3 (maximum positive impact). Thus, a score of 3 indicates the device made the individual to feel much more, for example, competent or embarrassed, than they feel without the device. A score of 0 indicate the device has no perceived impact, and a score of −3 is used when the individual feels a lot less (e.g. embarrassed/competent) when using the device.

PIADS has been shown to have good psychometric qualities [17] and clinical relevance [19], and PIADS has previously been used in studies investigating the effect of EGAT in children with complex needs [3,27].

### Procedure

The present study was conducted one year after data collection for the total Swedish population survey study was completed [6], and participants that had agreed to participate in an additional telephone interview in the survey were contacted by a research assistant. The research assistant was an experienced occupational therapist who contacted the participants by e-mail or telephone and scheduled a structured interview using The Psychosocial Impact of Assistive Devices Scale (PIADS). Participants could choose to respond to the interview by telephone or by e-mail. Adult participants responded via an assistant/relative (*n* = 18), by themselves (*n* = 10), by using a speech synthesizer that converted their written answers to speech (*n* = 2), or by e-mail (*n* = 4). PIADS interviews concerning child participants were conducted by telephone with the child’s parents. Informed consent for participation was obtained, for participants who responded by telephone it was obtained verbally and for participants who responded by e-mail it was obtained writing. The interview by telephone was held at the time of the day most convenient for the participant. The research assistant read the items out loud, one by one. The participants gave their perception of the psychosocial impact of the eye-gaze assistive technology on their everyday life and a rating for each item. If participants asked for clarifications on items, the research assistant read out loud from the PIADS glossary. The research assistant filled in the ratings on the PIADS scoring sheet. The interviews took approximately 30 min. Participants that preferred to respond by e-mail received an adapted PIADS scoring sheet. The adapted PIADS scoring sheet was a Word document with the 26 items and ratings of each item. The participant could fill in their ratings in the Word document and e-mail their answers to the research assistant.

### Data analysis

A response analysis was conducted to examine whether there were any differences between the participants in the total population study (TPS) and the present study. Analyses were conducted with chi-square (analyses of gender, diagnoses, use of EGAT in school/at work, and use of EGAT in leisure), and Mann-Whiney *U* tests (analyses of age, access to EGAT in months, total QUEST score). The level of significance was set at *p* ≤ 0.05.

The diagnoses reported in the population survey study are, in the present study, categorized into five diagnostic groups; cerebral palsy, stroke/multiple sclerosis, Rett syndrome, musculus dystrophy, or other (e.g. autism, spinal cord injury, amyotrophic lateral sclerosis), based on the frequency of occurrence. Descriptive statistics were calculated with means and standard deviations reporting participants’ age and years of access to eye-gaze assistive technology, and the total Quebec User Evaluation of Satisfaction with Assistive Technology (QUEST) score. The use of EGAT is categorized into four groups based on the duration of use per day: (1) “none user”, or someone who does not currently use EGAT; (2)“low duration user”, or someone who uses EGAT for two or fewer hours; (3)“medium duration user”, uses EGAT for more than two hours and up to four hours, and (4) “high duration user” who uses EGAT for more than 4 h.

The PIADS data was analysed and calculated for each of the three subscales, and conducted according to the PIADS manual in which embarrassment, frustration, and confusion are treated separately. Analyses are also conducted for each of the 26 PIADS items, and presented in mean values. To examine whether there were any differences between the adult and child participants, analyses were conducted with the Mann-Whitney *U* test (subscales and item-level). A significance level of *p* ≤ 0.05 was used. The psychosocial impact in relation to duration of use was investigated by comparing means, standard deviations, and ranges of mean values in the PIADS subcategories. Analyses were also conducted with the Kruskal-Wallis test (subscales and duration of use). A significance level of *p* ≤ 0.05 was used.

IBM Statistical Package for the Social Sciences version 28 (SPSS, Chicago, Illinois) was used in all statistical analyses.

## Results

### Response analyses

The response analysis between participants in the present study (*N* = 61) and participants in the TPS (*N* = 171) showed significant differences in age (mean =26 versus 31) (Mann-Whiney *U* test *p* = 0.002) demonstrating that the participants in the current study had a higher percentage of child participants than in the TPS; 44% versus 35%. There were fewer participants with the diagnosis of amyotrophic lateral sclerosis (ALS) in the present study (6.6% vs 17%) (chi-square *p* = <0.01) and significantly more participants used EGAT at work (36% versus 19%) (Chi-Square *p* = <0.03) and in leisure, 90% versus 55% (Chi-Square *p* = <0.01) compared to the TPS. No significant differences were found regarding gender, access to an EGAT, use of EGAT in school, or total QUEST score.

### Demographics of participants

The participants’ ages ranged from 5–74 years. Adults’ ages ranged from 19–74 years (mean =36, SD 15.5 years) and children’s ages ranged from 5–20 years (mean =13, SD 4.3 years). Of the 61 participants were 49% female (*n* = 30). The most common diagnosis was cerebral palsy (*n* = 32, 52%), followed by stroke/multiple sclerosis (*n* = 8, 13.1%), Rett syndrome (*n* = 7, 11.5%), Musculus dystrophy (*n* = 6, 9.8%) or other (e.g. autism, spinal cord injury, amyotrophic lateral sclerosis) (*n* = 8, 13.1%). Participants had had access to EGAT for more than three years (mean =39 months, SD 19). EGAT devices used by the participants were Tobii I–12 or Tobii I–15 (adults *n* = 16, 47%; children *n* = 20, 74%), Tobii PCEye or Tobii PCEye Go (adults *n* = 14, 41%; children *n* = 5, 19%) or other (Tobii C12, Intelligaze Rolltalk or Grid Pad Eye 13”) (adults *n* = 4, 12%; children *n* = 2, 7%). Participants communicated with other people in the same room by using EGAT (adults *n* = 22 65%; children *n* = 21, 78%), often in combination with other communication modalities, i.e. speech (adults *n* = 17, 50%, children *n* = 4, 15%), communication boards (adults *n* = 14, 41%, children *n* = 24, 89%), or speech application/speech synthesizer (adults *n* = 2, 6%, children *n* = 2, 7%). The EGATs were used during leisure time by almost every participant (all *n* = 56, 92%; adult *n* = 31, 91%; children *n* = 25, 92%), and several also used the EGAT at work/school (all *n* = 46, 75%; adult *n* = 24, 70%; children *n* = 22, 81%).

About one-third of the participants (*n* = 19, 31%) were categorized as “high duration users” of the EGAT. “Medium duration users” comprised *n* = 15 (25%) of the participants, and “low duration users” comprised *n* = 25 (41%) of the users. Two participants reported that they did not currently use the EGAT.

### Psychosocial impact

#### Analyses of subscales

The subscale of competence had the highest values for all participants (*N* = 61), (mean =1.63, SD 0.71). It was also the subscale with the highest value for adult participants (*n* = 34) (mean =1.76, SD 0.83). Children (*n* = 27) had the highest value in the adaptability subscale (mean =1.52, SD 0.54). The competence subscale was the only subscale with a significantly higher value for adult participants (*p* = 0.038) compared to children (Table 2).

**Table 2.** Display the three subscales and mean values of the ratings from all participants, adults and children. Group comparison between adults and children.

Subscale	Participants						Group comparison
	All *N* = 61		Adult *n* = 34		Children *n* = 27		
	Mean	SD	Mean	SD	Mean	SD	*P*
Competence^a^	**1.63**	0.71	**1.76**	0.83	1.48	0.50	0.038*
Adaptability^b^	1.56	0.75	1.60	0.88	**1.52**	0.54	NS
Self-esteem^c^	1.47	0.66	1.45	0.81	1.50	0.41	NS

^a^adequacy; performance; skilfulness; efficiency; usefulness; independence; productivity; capability; quality of life; competence and expertise.

^b^willingness to take chances; well-being; take advantage of opportunities; ability to participate; adapt to daily living and eagerness to try new things.

^c^security; confusion; self-confidence; self-esteem; happiness; embarrassment; frustration; sense of power and sense of control.

#### Analyses of items for all participants

The mean values of the participants’ (*N* = 61) rating for each item are presented in Table 3. Overall, the result from the ratings demonstrated that the EGAT had a positive psychosocial impact on participants’ everyday life, as demonstrated in Table 3. The three items with the most positive impact on all participants represented all three subscales; namely, quality of life (mean =2.28), ability to participate (mean =2.25), and self-esteem (mean = 2.08).

**Table 3.** Display the items included in PIADS. Mean values of the ratings from all participants, adults and children, and a group comparison between adults and children.

Items included in PIADS	All *N* = 61	Adult *n* = 34	Children *n* = 27	Group comparison
*The Subscales are reported with capital letters in brackets: Competence (C), Adaptability (A), Self-esteem (S)*	Mean	Mean	Mean	*p*
Quality of life (C)	2.28	2.24	2.33	NS
Ability to participate (A)	2.25	1.94	2.63	**0.050** *
Self-esteem (S)	2.08	1.94	2.26	NS
Wellbeing (A)	2.02	2.09	1.93	NS
Capability (C)	2.00	1.94	2.07	NS
Happiness (S)	1.97	2.03	1.89	NS
Competence (C)	1.87	1.85	1.89	NS
Sense of control (S)	1.84	1.85	1.81	NS
Self-confidence (S)	1.79	1.65	1.96	NS
Productivity (C)	1.74	1.94	1.48	NS
Performance (C)	1.72	1.76	1.67	NS
Expertise (C)	1.72	1.68	1.78	NS
Skilfulness (C)	1.70	1.71	1.70	NS
Independence (C)	1.70	1.91	1.44	**0.048** *
Usefulness (C)	1.56	1.88	1.15	**0.012** *
Sense of power (S)	1.54	1.50	1.59	NS
Eagerness to try new things (A)	1.46	1.50	1.41	NS
Efficiency (C)	1.41	1.97	0.70	**0.001** *
Adapt daily living (A)	1.36	1.38	1.33	NS
Take advantage of opportunities (A)	1.36	1.47	1.22	NS
Security (S)	1.30	1.38	1.19	NS
Willingness to take chances (A)	0.79	0.94	0.59	NS
Adequacy (C)	0.75	0.88	0.59	NS
Embarrassment (S)	−0.34**	−0.24**	−0.48**	NS
Frustration (S)	−0.70**	−0.65**	−0.78**	NS
Confusion (C)	−0.75**	−0.62**	−0.93**	NS

*display items with a significant difference between adult and children participants.

**negative scoring has a positive psychosocial impact.

#### Differences between adult and child users

The three items with the most positive impact for adults (*n* = 34) were: quality of life (mean =2.24), well-being (mean =2.09), and happiness (mean =2.03), For children (*n* = 27), the three items with the most positive impact were: ability to participate (mean =2.63), quality of life (mean =2.33), and self-esteem (mean =2.26); for detailed information, see Table 3. When conducting group comparisons, significant differences between adult and children’s participants were found in four items. Adults reported a higher psychosocial impact than children on the items: independence, (*p* = 0.048), usefulness (*p* = 0.012), and efficiency (*p* = 0.001), while children had a higher psychosocial impact on the item: ability to participate (*p* = 0.050).

### Duration of use

Table 4 shows the results from categorization of duration of EGAT use in both adults and children. The table demonstrates that more than half of adult users are high (44%) or medium (29%) users. Only a few children are categorized as high users (15%), while about two-thirds of the children (63%) are categorized as low users. Two participants (3%), one adult and one child user, did not use the EGAT at the time of the interview.

**Table 4.** Categorization of users based on the duration of use.

	All (*N* = 61)	Adult (*n* = 34)	Children (*n* = 27)
	N(%)	*n*(%)	*n*(%)
High duration user *(more than 4 h/day)* A (*n* = 10), B (*n* = 9), C (*n* = 0)^a^	19(31)	15(44)	4(15)
Medium duration user *(more than two hours and up to four hours/day)* A (*n* = 7), B (*n* = 7), C (*n* = 1)^a^	15(25)	10(29)	5(18)
Low duration user *(two or fewer hours/day)* A (*n* = 17), B (*n* = 3), C (*n* = 5)^a^	25(41)	8(24)	17(63)
None user A (*n* = 2), B (*n* = 0), C (*n* = 0)^a^	2(3)	1(3)	1(4)

^a^Used eye gaze devices, A (Tobii I-12 or Tobii I-15), B (Tobii PCEye or Tobii PCEye Go) and C (Tobii C12, Intelligaze Rolltalk or Grid Pad Eye 13”).

Based on the categorization of duration of use (high-, medium-, low duration or ‘none’ users), the participants’ mean values, standard deviations, and range of mean values are displayed in Table 5, for each of the subscales competence, adaptability and self-esteem.

**Table 5.** Mean values, standard deviation, and range in mean values in each of the subcategories competence, adaptability, and self-esteem.

	Competence	Adaptability	Self-esteem
	Mean (SD)	Mean (SD)	Mean (SD)
	mean range	mean range	mean range
High duration user	**1.78** (0.70)	1.58 (0.85)	1.5 (0.65)
	0.33 − 2.92	−0.33 − 2.83	0.38 − 3.00
Medium duration user	**2.01** (0.51)	**2.01** (0.62)	1.59 (0.77)
	1.17 − 2.75	1.00 − 3.00	0.38 − 2.75
Low duration user	1.41 (0.61)	1.41 (0.48)	**1.47** (0.52)
	0.17 − 2.42	0.50 − 2.33	0.25 − 2.38
None user	0.08 (0.71)	−0.17 (0.23)	0.31 (0.80)
	−0.42 − 0.58	−0.33 − 0.00	−0.25 − 0.88

In PIADS, high, medium and low duration users rated that EGAT has a positive impact on each of the three subscales. The none users have assesed that EGAT has a lower psycosocial impact—close to zero—compared with high, medium and low duration users.

**Figure 1.**
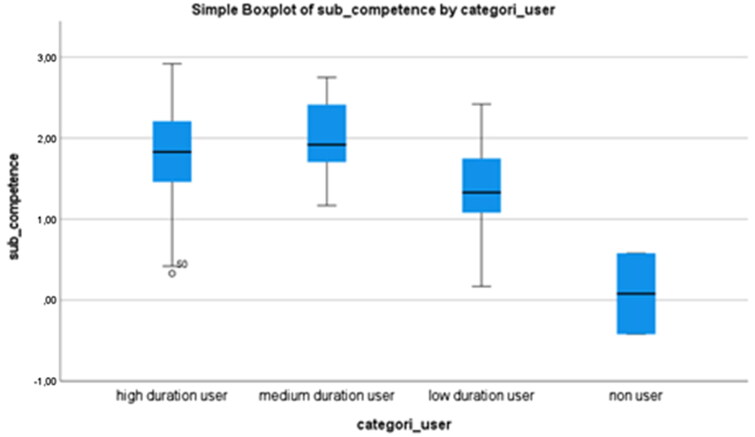
Boxplot displaying a variation of the impact of eye-gazed assistive technology on the subscale of competence based on the duration of use.

**Figure 2.**
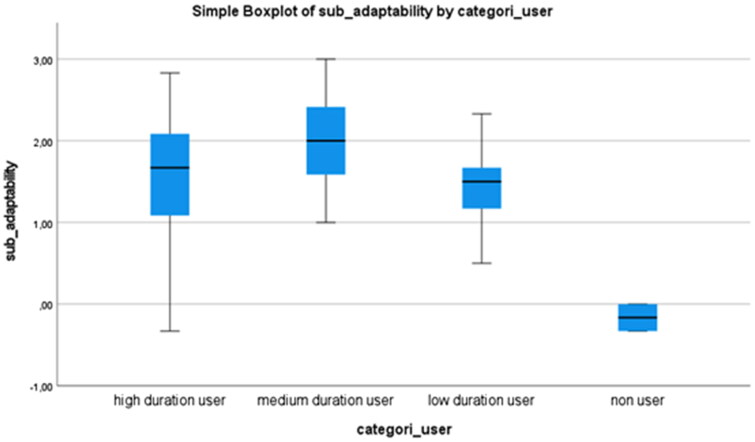
Boxplot displaying a variation of the impact of eye-gazed assistive technology on the subscale of adaptability based on the duration of use.

**Figure 3.**
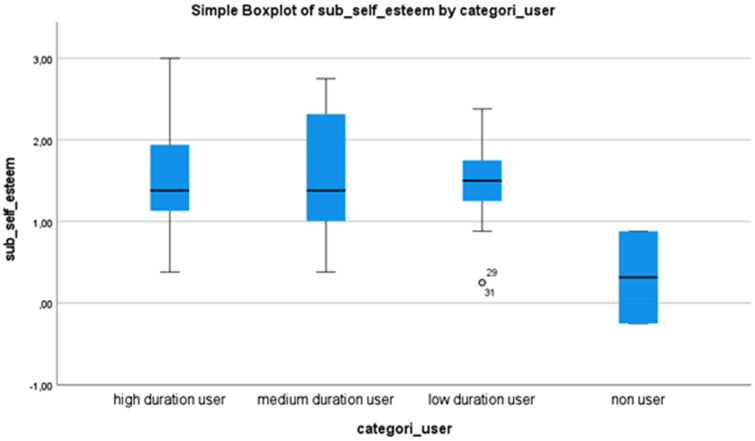
Boxplot displaying the variation of impacts of eye-gazed assistive technology on the subscale of self-esteem based on the duration of use.

Figures 1, 2 and 3 are boxplots that display mean values and the range of mean values that are reported in Table 5. This shows EGAT’s variation of impact on the subscales competence, adaptability, and self-esteem based on duration of use or ‘none’ use. No significant difference was found regarding duration of use in either of the subscales.

## Discussion

Overall, the study shows positive psychosocial impact in competence, adaptability, and self-esteem among both adult and child users with severe disabilities, with a higher impact on competence for adults compared to children, and with the highest impact on psychosocial aspects; namely, quality of life, ability to participate, and self-esteem. Moreover, the study also indicates that the high psychosocial impact that was found is not necessarily related to duration of use, since both low-, medium- and high-duration users that participated in the study had about the same level of positive ratings. Hence, users may experience high psychosocial impacts from EGAT even with low duration of use in everyday life, which is important information for service providers. Altogether, almost all the participants in this study are EGAT users who have maintained their use for about three years. The high values of positive psychosocial impact may therefore mirror how beneficial the technology is for these individuals. Non-users who for some reason have stopped their use might report other values regarding psychosocial impact. A future study should investigate the psychosocial impact in a group who has abandoned the device, to better understand how psychosocial impact varies within the population eligible for the device.

The study adds new knowledge on EGAT outcomes to previous intervention research [3,9,28] and usability research [6] by expanding understanding on why users feel the technology is worth using. The results showed overall high positive psychosocial impacts of EGAT (subscales: mean =1.47–1.76; 3 items with highest impact: mean =2.03–2.63) are in line with the few previous studies that have been published. Positive psychosocial impacts from EGAT in children were also found in previous studies [3,7,27] when using EGAT in everyday life. Moreover, the high impact of the aspects *quality of life* (mean =2.28), *ability to participate* (mean =2.25) and *self-esteem* (mean =2.08) in the current study indicate important changes in participants’ perceptions regarding opportunities to participate in daily and community life. Based on the study’s results, EGAT seems to have benefits in daily life among users. Most EGAT users have severe physical disabilities, communication difficulties, and are dependent on others for everything they want to do or when communicating. The study results therefore show how EGAT makes them feel in terms of competence, adaptability, and self-esteem.

One interesting finding was that the positive psychosocial impact of *competence* showed significantly higher impact for adults than for children. According to [26], the competence subscale is sensitive to the perceived impact on performance and productivity in everyday activities. Higher impact for adults seems realistic and positive, since it may reflect that EGAT supports them in assuming their adult responsibilities to get things done and manage everyday life. In addition, the results also showed that among the four items that significantly differed in ratings between adults and children, the three items of *independence, efficiency,* and *usefulness* (all of which relate to the competence subscale), were higher for adults compared to children. Since the children’s ages ranged from 5 years (mean =13, SD =4.3) it is not surprising that the psychosocial impact from the device on usefulness, efficiency, and independence was higher for adults. Adults are supposed to be independent and efficient. Just as all children need to explore and learn activities at younger ages, research shows that children with severe disabilities that use EGAT need opportunities in everyday life to explore activities, and demonstrate competencies [5,11] and gaining experiences of eye-gaze performance [29,30] before effectively and independently managing activities of daily living. In addition, gradually becoming more independent is part of development and personal growth for all children [31]. Previous intervention research among child users also indicates that feelings of competence may increase over time due to becoming more experienced users of EGAT [3], which is promising.

The results also showed the highest psychosocial impact for the aspect *ability to participate* for children. Parents’ perceived high impact on their child’s ability to participate is very hopeful, since participation is crucial to support children’s development, learning, and well-being [31,32]. Research shows that children with complex needs face restricted social participation and often are involved in few activities [33,34]. The high impact on the children’s ability to participate corresponds with previous intervention studies that showed that EGAT can increase children’s communicative interaction and activity repertoire in daily life [3,9].

High-, medium- and low-duration users were included in this study, with most adults being high- or medium-duration users (73%), and the majority of participating children being low-duration users (63%). This shows that EGAT’s psychosocial impact doesn’t relate to how many hours the technology is used in everyday life. Even low-duration users, with up to 2 h of use a day, which is especially common among children [3,6], may perceive an important psychosocial impact on everyday life from using the device. The results with boxplots on the two participants with non-use at the moment of data collection, who had low ratings in PIADS, validate the data by showing the discrepancy in ratings between individuals using and not using the device. Therefore, clinicians should consider how to evaluate the user’s positive and negative feelings when using EGAT. Service providers need also to be aware of that even a low-duration user may experience major positive changes in psychosocial impact with EGAT compared to not having access to the device in daily life. This is important to consider for service providers when evaluating the use of AT at follow-up, to more fully understand the usability and the retention or abandonment of EGAT.

This study indicates the useworthiness of EGAT in daily life, as the results show how users feel it impacts them personally, regardless of whether they are a high- or low-duration user [17] has shown that higher positive scores in PIADS seem to be associated with adoption and retention of an assistive technology. For example, a study [20] that used PIADS to investigate the psychosocial impact of hearing aids on hard of hearing people found one year later that individuals that had adopted the device had shown high values on all subscales (1.5 or more on average), and those that abandoned it had low values (<1.00). The results with low ratings (<1.00) of the two participants in the current study that no longer used the EGAT are in line with this result. The current study therefore indicates that the positive psychosocial feelings among users may be one explanation for the uptake and retention of EGAT in daily life.

The study provides clinically relevant knowledge of considering psychosocial aspects of EGAT to understand whether and why it is worth using EGAT in daily life despite low- or high-duration EGAT use. Adding socioemotional aspects to functional and technical aspects when analysing the use of a device such as EGAT will help the field understand success and adversity regarding EGAT use for individuals. To capture users’ own feelings about EGAT, an instrument that measures personal perceptions, like PIADS, is useful. The study also provides knowledge for decision-makers and for those who decide on EGAT guidelines, as well as information to parents about which psychosocial aspects may improve by using EGAT in daily life.

### Limitations

Compared to the survey study, the participants in the current study were found to be younger, there were more children included, fewer participants with ALS, and more adults used EGAT at work/school. Also, those from the survey study that agreed to participate, may have more positive feelings about EGAT use in everyday life and therefore were more interested in sharing their feelings and experiences. Generalizations to the whole population of EGAT candidates and users therefore should be done with caution, since the psychosocial impact may vary more widely in a larger population. Another consideration is that the PIADS interviews were conducted one year after the survey study. Demographic data is based on the survey study and may therefore not fully represent the actual users.

Interviewing participants with communication difficulties may be challenging. Using communication devices during a telephone interview may have limited these participants’ responses, depending on the effort required while using the assistive device. Despite these difficulties, it is important to interview individuals with communication difficulties instead of proxy respondents whenever possible to obtain important insights into their own perceptions and feelings [35], and to better understand how a device such as EGAT impacts daily life. With the structured nature of the PIADS, and with an occupational therapist experienced in communication difficulties carefully conducting the interviews, some of the potential threats to validity may have been overcome. Another concern is that parents answered on behalf of child participants, and research indicate the risk of subjectivity in responses from proxy respondents [35]. A future qualitative study is therefore needed to investigate child users’ own perceptions and perspective of the impact of EGAT in everyday life. For example, the lower duration of use among children doesn’t have to mean that they don’t want to use the device more, since environmental factors like children’s dependence on adults, may limit their use. A qualitative interview study with children would therefore clarify children’s own perceptions and perspective on psychosocial consequences when using or not being able to use EGAT.

Feelings of negative psychosocial consequences related to stigmatization are a common reason for non-use of an AT [36]. Based on the current study’s participants, all of whom were long-term users and willing to use the AT, it is not possible to exclude the possibility that EGAT may give rise to negative feelings of psychosocial impact and feelings of stigmatization, since non-users didn’t participate. A future study investigating the psychosocial impact of EGAT among those who have abandoned EGAT would add more knowledge to this topic.

## Conclusions

In conclusion, this study demonstrated that among long-term and maintained users of EGAT in everyday life, there are, above all, perceptions of high positive psychosocial impact on competence, adaptability, and self-esteem among both adults and children. The study adds new knowledge, in that high positive psychosocial impacts may be found regardless of the device’s duration of use, even among low-duration EGAT users, which is important for service providers to consider. The high impact shows that using EGAT, over time, results in positive feelings in different areas of the user’s life, indicating opportunities to participate in daily and community life which can explain the reasons for adopting the technology as an AT. A future study should focus on the psychosocial impact from EGAT on individuals that are assessed to be candidates of and have had opportunities to use EGAT but are not using the device. This would give insights about whether there are specific negative psychosocial factors associated with abandonment and stigmatization that need to be considered in service delivery. A qualitative study involving children would also lead to increased understanding about their own perceptions of the opportunities and psychosocial consequences of using or not using EGAT in their natural environments.

## Data Availability

The data that support the findings of this study are available from the corresponding author, [MA], upon reasonable request.
